# Current News

**Published:** 1900-01

**Authors:** 


### Current News.

### A BILL FOR THE APPOINTMENT OF DENTAL SURGEONS IN THE ARMY.

THE following bill was presented to the House of Representatives on December 5, 1899:

IN THE HOUSE OF REPRESENTATIVES, DECEMBER 5, 1899.

Mr. Otey introduced the following bill, which was referred to the Committee on Military Affairs and ordered to be printed:

*A Bill to provide for the Appointment of Dental Surgeons for Service in the United States Army.*

*Be it enacted by the Senate and House of Representatives of the United States of America in Congress assembled*, That the Surgeon-General of the Army, with the approval of the Secretary of War, be, and he is hereby, authorized to employ and appoint dental surgeons to serve the officers and enlisted men of the regular and volunteer army in the proportion of one dental surgeon to every one thousand of said army. Said dental surgeons shall be employed as contract dental surgeons, under the terms and conditions applicable to army contract surgeons, and shall be graduates of standard medical or dental colleges, trained in the several branches of dentistry, of good moral and professional character, and shall pass a satisfactory professional examination: *Provided*, That three of the number of dental surgeons to be employed shall be first appointed by the Surgeon-General, with the approval of the Secretary of War, with reference to their fitness for assignment, under the direction of the Surgeon-General, to the special service of conducting the examinations and supervising the operations of the others, and for such special service an extra compensation of sixty dollars a month shall be allowed: *Provided further*, That dental college graduates now employed in the Hospital Corps, who have been detailed for a period of not less than twelve months to render dental service to the army and who are shown by the reports of their superior officers to have rendered such service satisfactorily, may be appointed contract dental surgeons without examination.

---

### THIRTEENTH INTERNATIONAL MEDICAL CONGRESS.

BALTIMORE, MD., November 1, 1899.

THE American National Committee of the Thirteenth International Medical Congress, to be held in Paris from the 2d to the 9th of August, 1900, in connection with the French Exposition, has been organized as above indicated.

All Doctors of Medicine are entitled to membership in this Congress by making the proper application and paying the sum of five dollars. The Secretary-General in Paris has instructed the American National Committee to receive the applications of American physicians, and for this purpose a blank form is enclosed, upon which is to be written full name and address, degrees, and any position of note held, together with the section of the Congress to which the writer wishes to belong. A visiting-card should also be appended. These forms, with the five dollars, are to be returned to the Secretary of the National Committee. He in turn will send receipt and forward the slips and money to Paris, where they will be registered, and in due course of time a card of admission to the Congress will be mailed to each applicant.

The Committee hopes the American representation in this extremely important Medical Congress may be as large as possible, and they would urge every member of the profession to enter his name for membership, this alone entitling him to receive a digest of the full proceedings of the Congress and the printed report[1] of the Section to which he belongs.

The Sections are as follows:

### CLASS I.

#### BIOLOGICAL SCIENCES.

A. Section of Descriptive and Comparative Anatomy. Secretary, M. Auguste Pettit, 60, rue Saint-André-des-Arts, Paris.

B. Section of Histology and Embryology. Secretaries, MM. Retterer and Loisel, 15, rue de l'Ecole-de-Médecine, Paris.

C. Section of Physiology, and Biological Physics and Chemistry. Secretary, M. Dastre, à la Sorbonne, Paris.

### CLASS II.

#### MEDICAL SCIENCES.

A. Section of General Pathology and Experimental Pathology. Secretaries, M. Charrin, 11, avenue de l'Opéra, Paris; M. Roger, 4, rue Perrault, Paris.

B. Section of Bacteriology and Parasitology. Secretary, M. R. Blanchard, 226, boulevard Saint-Germain, Paris.

---

[1] Communications respecting the delivery of these reports to members to be addressed to M. Masson, publisher, of the proceedings of the Congress, 120, boulevard St-Germain, Paris.

C. Section of Pathological Anatomy. Secretary, M. Letulle, 7, rue de Magdebourg, Paris.

D. Section of Internal Pathology. (General Medicine.) Secretaries, M. Rendu, 28, rue de l'Université, Paris; M. Widal, 155, boulevard Haussmann, Paris.

E. Section of Medicine of Infancy. (Diseases of Children.) Secretary, M. Marfan, 30, rue La Boëtie, Paris.

F. Section of Therapeutics, Pharmacology, and Materia Medica. Secretary, M. Gilbert, 27, rue de Rome, Paris.

G. Section of Neurology. Secretary, M. P. Marie, 3, rue Cambacérès, Paris.

H. Section of Psychiatry. Secretary, M. Ant. Ritti, Asile de Charenton, Seine (France).

I. Section of Dermatology and Syphilography. Secretary, M. G. Thibierge, 7, rue de Surènes, Paris.

### CLASS III.

### SURGICAL SCIENCES.

A. Section of General Surgery. Secretary, M. Walther, 21, boulevard Haussmann, Paris.

B. Section of Surgery of Infancy. Secretaries, M. A. Broca, 5, rue de l'Université, Paris; M. Villemin, 58, rue Notre-Dame-des-Champs, Paris.

C. Section of Urinary Surgery. Secretary, M. Desnos, 31, rue de Rome, Paris.

D. Section of Ophthalmology. Secretary, M. Parent, 26, avenue de l'Opéra, Paris.

E. Section of Laryngology and Rhinology. Secretary, M. Lermoyez, 20 bis, rue La Boëtie, Paris.

F. Section of Otology. Secretary, M. Castex, 30, avenue de Messine, Paris.

G. Section of Stomatology. Secretary, M. Ferrier, 39, rue Boissy-d'Anglas, Paris.

### CLASS IV.

### OBSTETRICS AND GYNÆCOLOGY.

A. Section of Obstetrics. Secretaries, M. A. Bar, 122, rue La Boëtie, Paris; M. Champetier de Ribes, 28, rue de l'Université, Paris.

B. Section of Gynæcology. Secretary, M. Hartmann, 4, place Malesherbes, Paris.

CLASS V.

PUBLIC MEDICINE.

A. Section of Legal Medicine. Secretary, M. Motet, 161, rue de Charonne, Paris.

B. Section of Military Surgery and Medicine. Secretary, M. Catteau, Ministère de la Guerre, Paris.

Members desiring to present papers will forward the title and a *résumé* before May 1, 1900, to the secretary of the section to which they belong, for each sectional committee reserves to itself the right of drawing up its own working programme. Papers are limited to fifteen minutes.

HENRY BARTON JACOBS,
*Secretary American National Committee.*

### KENTUCKY STATE DENTAL ASSOCIATION.

THE Annual Meeting of the Kentucky State Dental Association will be held in the city of Louisville, on the 15th, 16th, and 17th of May, 1900. We are already assured of the best meeting in the history of the Association. Aside from an attractive programme, the meeting of the National Confederate Association in Louisville at the same time enables us to procure a one cent per mile railroad rate from over the greater portion of the United States. There will be many other attractions to the dentists who attend,—trips to the wonderful Mammoth Cave and to the blue-grass regions of Kentucky.

Ample accommodations at reasonable rates have already been obtained.

For further information, address

F. I. GARDNER, D.D.S.,
*Secretary.*

213 WEST CHESTNUT STREET, LOUISVILLE, KY.

### NEW YORK ODONTOLOGICAL SOCIETY.

THE Thirty-second Anniversary of the above Society will take place at the Academy of Medicine, Tuesday, January 16, 1900, at two and eight P.M.

At the afternoon session, commencing at two o'clock, Dr. Joseph Head, of Philadelphia, will give a clinic,—Inserting a Porcelain Inlay, using an entirely new cement. Immediately after the clinic Dr. Head will read a paper: subject, "Shadow Problems as presented by Porcelain Inlays."

The evening paper, by A. W. Harlan, M.D., D.D.S., of Chicago: subject, "A Review of Recent Literature on the Loose-Tooth or Pyorrhœa Problem."

*Executive Committee.*—Dr. W. W. Walker, *Chairman*, 58 West Fiftieth Street; Dr. C. B. Nash, Dr. F. T. Van Woert.

---

### SECOND DISTRICT DENTAL SOCIETY, STATE OF NEW YORK.

A REGULAR meeting of the Second District Dental Society of the State of New York, at which the First District Dental Society and the Central Dental Association of Northern New Jersey will be present as guests, will be held at "The Argyle," 308 Fulton Street, opposite Johnson Street, Monday evening, January 8, 1900.

Paper: "The Soft Tissues about the Teeth: Their Morphology and Pathology," by Dr. I. Norman Broomell, Philadelphia. The paper will be followed by a series of original lantern slides pertaining to the subject.

The names of the following applicants for membership, having been received and endorsed by the Censors, will be balloted upon: Dr. Frank S. Ketcham, 359 Macon Street; Dr. S. S. Keowen, 949 Bedford Avenue; Dr. Frank Sanbern, 287 Jefferson Avenue; Dr. W. B. Dills, 260 DeKalb Avenue.

F. B. KEPPY,
*Recording Secretary.*

62 HANCOCK STREET, BROOKLYN, N. Y.

